# Systematic review of SLC4A11, ZEB1, LOXHD1, and AGBL1 variants in the development of Fuchs’ endothelial corneal dystrophy

**DOI:** 10.3389/fmed.2023.1153122

**Published:** 2023-06-27

**Authors:** Tatiana Romanovna Tsedilina, Elena Sharova, Valeriia Iakovets, Liubov Olegovna Skorodumova

**Affiliations:** ^1^Laboratory of Human Molecular Genetics, Lopukhin Federal Research and Clinical Center of Physical-Chemical Medicine of Federal Medical Biological Agency, Moscow, Russia; ^2^I.M. Sechenov First Moscow State Medical University, Moscow, Russia

**Keywords:** Fuchs dystrophy, SLC4A11, ZEB1, LOXHD1, AGBL1, posterior polymorphous corneal dystrophy type 3, variants, mutations

## Abstract

**Introduction:**

The pathogenic role of variants in TCF4 and COL8A2 in causing Fuchs’ endothelial corneal dystrophy (FECD) is not controversial and has been confirmed by numerous studies. The causal role of other genes, SLC4A11, ZEB1, LOXHD1, and AGBL1, which have been reported to be associated with FECD, is more complicated and less obvious. We performed a systematic review of the variants in the above-mentioned genes in FECD cases, taking into account the currently available population frequency information, transcriptomic data, and the results of functional studies to assess their pathogenicity.

**Methods:**

Search for articles published in 2005–2022 was performed manually between July 2022 and February 2023. We searched for original research articles in peer-reviewed journals, written in English. Variants in the genes of interest identified in patients with FECD were extracted for the analysis. We classified each presented variant by pathogenicity status according to the ACMG criteria implemented in the Varsome tool. Diagnosis, segregation data, presence of affected relatives, functional analysis results, and gene expression in the corneal endothelium were taken into account. Data on the expression of genes of interest in the corneal endothelium were extracted from articles in which transcriptome analysis was performed. The identification of at least one variant in a gene classified as pathogenic or significantly associated with FECD was required to confirm the causal role of the gene in FECD.

**Results:**

The analysis included 34 articles with 102 unique ZEB1 variants, 20 articles with 64 SLC4A11 variants, six articles with 26 LOXHD1 variants, and five articles with four AGBL1 variants. Pathogenic status was confirmed for seven SLC4A11 variants found in FECD. No variants in ZEB1, LOXHD1, and AGBL1 genes were classified as pathogenic for FECD. According to the transcriptome data, AGBL1 and LOXHD1 were not expressed in the corneal endothelium. Functional evidence for the association of LOXHD1, and AGBL1 with FECD was conflicting.

**Conclusion:**

Our analysis confirmed the causal role of SLC4A11 variants in the development of FECD. The causal role of ZEB1, LOXHD1, and AGBL1 variants in FECD has not been confirmed. Further evidence from familial cases and functional analysis is needed to confirm their causal roles in FECD.

## 1. Introduction

Fuchs’ endothelial corneal dystrophy (FECD) is a bilateral primary inherited eye disease associated with a gradual loss of the corneal endothelial cells (CEnCs) (1, 2). The formation of excrescences on the thickened Descemet membrane - guttae - is a characteristic sign of FECD (3). The main function of the CEnCs is to maintain the water balance in the corneal stroma (4). As the number of CEnCs decreases, they are unable to pump water out of the corneal stroma and prevent excessive aqueous humor flow from the anterior chamber and corneal edema develops. The progression of FECD is associated with the vascularization and fibrosis of the cornea. This results in the loss of visual acuity down to the point of light perception.

FECD is the most common primary corneal endothelial dystrophy but the prevalence varies between populations. Epidemiologic studies indicate that FECD is more prevalent in Europe and the United States than in Asian populations. For example, in Iceland, guttae were found in 11% of women and 7% of men over the age of 55 (5). In other European and American population studies, FECD was found in 3.9–5.2% of the population over the age of 40 (1, 6, 7). In a Japanese population study of 107 cataract patients, four cases of FECD (3.7%) were identified (8). Another study compared the incidence of FECD between Chinese Singaporeans and Japanese (9). It was found that FECD was significantly more common in Singapore: 8.5% vs. 5.5% in Japan. A recent meta-analysis that includes the above studies showed a pooled prevalence estimate of 7.33% (10).

Clinically, FECD can be divided into early-onset and late-onset forms. The early-onset form manifests clinically in the second to third decades of life and has an autosomal-dominant mode of inheritance. Magovern was the first to describe a four-generation family with atypical histopathologic changes and the onset of symptoms in childhood (11). Biswas and co-authors investigated two other families with early onset of the disease. For the first time, they identified the Gln455Lys variant in the COL8A2 gene in patients with a family history of FECD and posterior polymorphous corneal dystrophy subtype 3 (PPCD3) (12). In general, this form is rare, although several cases have been described (13–17). In the predominant, late-onset form of FECD, the symptoms develop after the age of 50 years, with a global meta-analysis reporting a mean age of 61.9 (95% CI: 58.8–65.2) (1, 10, 18, 19). Autosomal-dominant inheritance has been established for late-onset FECD (1, 18–21).

Since the first studies of FECD, the preponderance of women over men was noted (22). This has been confirmed in the later studies, although the ratio has varied from 1.05 to 3.7:1, but in all cases women have been predominant (1, 5–7, 19, 23). It is worth mentioning that in a large family with early-onset FECD, the female-to-male ratio was 1:1 (11).

The methods used to study the etiology of FECD are diverse and changing with technological advances. FECD is a genetic disease; up to 50% of clinical cases are familial, and large families with dominant inheritance of the disease have been described (1, 11). The first method used to study the genetics of FECD was linkage analysis. Probands and relatives in previously clinically described families were investigated. This led to the discovery of several loci, but only a few were refined down to the coordinates of the variants. The loci defined in linkage analyses have been used to detect early-onset FECD candidate variants in the COL8A2 gene and variants in LOXHD1, AGBL1, and ZEB1 genes (12, 13, 24–26). Later, genome-wide association studies (GWASs) were performed in cohorts of FECD patients (23, 27, 28). GWASs were efficient in detecting the association of FECD with variants in the TCF4 gene (27). The association of FECD with loci in KANK4, LAMC1, and ATP1B1 genes has been reported based on GWAS results (28). The next widely used method to study the genetics of FECD is Sanger sequencing. It has been used to genotype the single variants in replication studies, and whole-gene sequencing has been used to find new candidate variants. For example, Biswas and co-authors sequenced coding exons of the COL8A2 gene in all affected and unaffected members of a family with early-onset FECD to find the causal mutation (12). Riazuddin’s team searched for variants in the SLC4A11 gene by sequencing all coding regions in FECD patients (29). Massive parallel sequencing was also introduced in genetic studies of FECD as it became affordable. A custom capture panel was used in the study by Wieben et al. to establish the absence of a single causative variant for FECD in the TCF4 gene (30). Exome sequencing has been used to detect variants in LOXHD1 and AGBL1 genes at previously identified loci (24, 25). A potentially pathogenic variant in the TSPOAP1 gene was discovered in the transcriptome data (31).

As a result, several genes have been implicated in the development of FECD. In recent reviews, TCF4, SLC4A11, ZEB1, COL8A2, LOXHD1, and AGBL1 genes have been repeatedly mentioned as being involved in the genetics of FECD (2, 32–37). Below, we briefly describe the genes harboring variants whose contribution to the etiology of FECD has been confirmed by segregation studies in families and by functional analysis.

The pathogenicity of COL8A2 variants NP_005193.1:p.Leu450Trp, NP_005193.1:p.Gln455Lys, and NP_005193.1:p.Gln455Val in the early-onset FECD patients has been confirmed by the results of genetic and molecular studies in native specimens and model systems (13, 38–42). Although there were early-onset cases with no identified variants in the coding exons and the second intron of the COL8A2 gene, the presence of single-nucleotide variants (SNVs), insertions and deletions, or copy number variations (CNVs) in the non-coding exon and the first intron was not excluded (43).

The association of TCF4 variants with late-onset FECD (especially in European descent populations) has been discovered by GWAS and confirmed by dozens of case–control studies (27). The single-nucleotide polymorphism (SNP) rs613872 in the TCF4 gene is the most studied association marker of the late-onset FECD and its association was significant in several studies and in the meta-analysis (30, 44–56). The CTG18.1 trinucleotide repeat expansion in the TCF4 gene has been detected in a large proportion of late-onset FECD patients and segregated in familial cases, so it is currently considered a causal variant (30, 45, 48, 50, 51, 55, 57–64). Its discovery by Wieben and co-authors was a breakthrough in understanding the genetics of late-onset FECD (57). The pathogenic mechanisms of CTG18.1 trinucleotide repeat expansion-associated FECD have recently been comprehensively reviewed by Fautsch and co-authors (35). Briefly, the mechanisms investigated to date are repeat-associated RNA toxicity, repeat-associated non-AUG translation, and dysregulation of TCF4 expression (31, 61, 65–73).

In summary, the presence of pathogenic variants in the COL8A2 and TCF4 genes has been comprehensively demonstrated, although further investigation of unresolved familial cases and pathogenic mechanisms is warranted (35). Thus, we have chosen not to review variants in these genes here. The role of other genes, SLC4A11, ZEB1, LOXHD1, and AGBL1, in FECD genetics seems more complicated and less obvious. We found a systematic review of SLC4A11 variants (74). The association of the c.1195G > A variant with FECD was evaluated by meta-analysis. We had less stringent selection criteria, which allowed us to include more records and not limit the analysis to meta-analysis. We did not find any meta-analyses or systematic reviews on the variants identified in ZEB1, LOXHD1, and AGBL1 in FECD patients.

The search for variants in FECD has been going on for many years, but there has not been a comprehensive reevaluation of the pathogenicity of variants in terms of the ACMG guidelines (75). In brief, the frequency of a variant in the population is important in the assessment of the pathogenicity of a variant. The effect of the variant on the protein is also taken into account: loss-of-function variants are considered a very strong criterion for the presence of a pathogenic effect. Existing functional studies can also significantly influence assessing pathogenicity. Family case studies, especially the segregation of the variant with the phenotype, are important not only for assessing pathogenicity but also for confirming the causal role of the variant. If the variant identified in the proband is absent in another first-degree relative with FECD, this is a strong argument against the causal role of the variant. One element of evaluation that is not considered in the ACMG criteria is the presence of gene expression in the tissue affected by the disease or associated with its pathogenesis. The expression of the gene may be assumed as a given, however, this is not always the case. Therefore, we considered it necessary to perform a systematic review of the variants in the SLC4A11, ZEB1, LOXHD1, and AGBL1 genes in the FECD, taking into account the currently available population frequency information, transcriptomic data, and the results of functional studies to assess their pathogenicity.

## 2. Methods

This systematic review was performed in compliance with the Preferred Reporting Items for Systematic Reviews and Meta-Analyses (PRISMA) guidelines.

### 2.1. Eligibility criteria

For the systematic review of variants, we included records selected according to the following criteria:

1. The record was published in a peer-reviewed journal as an original research article written in English, not as a review, abstract, poster, conference paper, or PhD thesis;
2. The article described genetic variants in Homo sapiens;
3. The article described variants identified in SLC4A11, AGBL1, LOXHD1, and ZEB1 genes in FECD or PPCD sporadic or familial cases, or functional experiments, including transcriptomic analysis in samples, harboring variants reported in these diseases.
4. The coordinates of the identified variants have been explicitly described, or the cDNA and/or protein coordinates of the aberration have been reported.

For information on the expression of the genes of interest, we searched the following articles:

1. The record was published in a peer-reviewed journal, an original research article written in English;
2. Gene transcription (SLC4A11, AGBL1, LOXHD1, or ZEB1) was examined in human corneal endothelial samples (control donor samples, FECD patient samples, PPCD patient samples, primary cultures, cell lines, human embryonic stem cell-derived corneal endothelium, and induced pluripotent stem cell-derived corneal endothelium);
3. Gene expression (SLC4A11, AGBL1, LOXHD1, or ZEB1) was evaluated in transcriptome data (RNA-seq, microarray expression analysis, single-cell RNA-seq, cDNA libraries sequencing, and CAGE sequencing), not PCR;
4. Gene expression defined from transcriptomic data was mentioned in the article text, figure, or supporting information.

### 2.2. Search methods

Search for articles on variants was performed manually from July 2022 to November 2022 and included articles published between 2005 and 2022. For AGBL1 and LOXHD1 genes, no studies of variants in FECD cases were found before 2012. Articles were initially searched by TT and VI, and independently by LS. TT and VI generated an “Initial pull 1” of articles from PubMed search results using the keywords: “gene (gene = ZEB1, LOXHD1, or AGBL1)” AND “variants”; “SLC4A11” AND “mutation.” LS generated “Initial pull 2” of articles from PubMed Central and Google Scholar search results by keywords: “gene (gene = SLC4A11, ZEB1, LOXHD1, or AGBL1)” AND “disease (disease = Fuchs OR PPCD).” The titles, abstracts, and full texts of articles from the initial pull were screened for compliance with the inclusion criteria. TT and VI also screened the reference lists of included articles from “Initial pull 1” and published reviews that appeared in the search results to identify additional relevant studies. Studies were grouped by genes of interest. After comparing “Initial pull 1” and “Initial pull 2” and removing duplicate articles, a “Final list of articles” of non-duplicate articles was created. LS screened the reference lists of articles from “Initial pull 2” that were missing from “Initial pull 1” to identify additional relevant studies and added them to the “Final list of articles (Supplementary Table S1).”

Search for articles on gene expression in transcriptome data was performed manually in February 2023, and included articles published between 2013 and 2022. The list of articles was generated by LS from Google Scholar search results using the keywords: “gene (gene = ZEB1, LOXHD1, or AGBL1) transcriptome analysis human corneal endothelium.” Titles, abstracts, and full texts of articles from the search results were screened for compliance with inclusion criteria. Articles meeting the inclusion criteria were included in the “List of articles expression (Supplementary Table S1).”

### 2.3. Article data extraction

TT, VI, and LS independently screened the abstracts and full texts of articles included in the “Final list of articles.” TT and VI performed the initial data extraction from the articles, while LS reviewed all of the data in the tables and edited or added missing information. We assessed the compliance of the data presented in the text and on the figures or tables. If there was a discrepancy between the raw data (including experimental data) and their interpretation in the text, we used the raw data to reassess pathogenicity. From each article, data (including experimental) for each detected variant were extracted and entered into the Gene_cases table, here and below Gene = SLC4A11, ZEB1, LOXHD1, or AGBL1. We indicated the diagnosis of the proband(s) (FECD or PPCD) included in each study. We also reported the ethnicity or country of residence of probands enrolled in each study (Supplementary Table S1). If available, information on the proband’s relatives, including their phenotype and genotype status, as well as their segregation, was entered into the Gene_familial_cases table (Supplementary Table S1). If segregation data were available for the variant, this was noted in the corresponding column of the Gene_cases table. If the functional analysis was conducted in the article, it was noted in the corresponding column of the Gene_cases table. The availability of information on the CTG18.1 repeats status in the carrier of the reported variant was noted in the corresponding column. In addition, the study design of the processed article was entered in the corresponding column of the Gene_cases table. If the processed article had a non-consecutive case series design or was a case report, the total number of alternative alleles in the probands and, if available, information on control group genotyping was entered. If the processed article had a consecutive case series design, information on the total number of unrelated probands screened and, if available, the number of probands with alternative alleles was entered in the appropriate columns. If the article was a case–control study, data on the total number of probands and controls screened, the number of probands and controls with alternative alleles, and the total number of alternative alleles in probands and controls were included. The minor allele frequency (MAF) was calculated for variants if more than 30 probands were tested. *p*-values for association tests were also reported if available.

From articles describing gene expression in the transcriptomic data, we extracted tissue type, cultivation status (*ex vivo* or different types of cultured cells), the technology used to generate transcriptomic data, and data on the expression of the SLC4A11, ZEB1, LOXHD1, or AGBL1 genes. This information is available in the Genes_expression_data table (Supplementary Table S1).

### 2.4. Variant description

For each described variant, rsID, HGSV genomic coordinate, reference allele, alternative allele, location of the variant in the gene region, the type of aberration, and variant effect were entered in the Gene_cases tables.

Variant’s rsIDs from articles have been checked in dbSNP for the up-to-date rsIDs and mistypes (76). We used the dbSNP database from July 2022 to November 2022. Variant description according to HGVS recommendations on genomic, transcript, and protein levels was identified from dbSNP (77). SLC4A11 transcript NM_032034.4, SLC4A11 isoform NP_114423.1, ZEB1 transcript NM_030751.6, and ZEB1 isoform NP_110378.3, AGBL1 transcript NM_152336.4 and AGBL1 isoform NP_689549.3, LOXHD1 transcript NM_144612.7, and LOXHD1 isoform NP_653213.6 were used the most in included articles.

If variants were described using transcript or protein sequence in the original article, we validated them and identified genomic coordinates through the Mutalyzer using NM or NP IDs mentioned in the article (78). All variant descriptions on transcript and protein levels were assigned to the same transcripts and isoforms for each gene mentioned above. If the resulting variant descriptions on transcript or protein levels differed from those in the original article, we noted this in the table.

The variants described in the Gene_cases tables have been summarized in the Gene_variants tables (Supplementary Table S1). They contain a list of unique variants in each gene. Each variant was described using rsID, if available, genomic coordinates, transcript, and protein changes according to the HGVS nomenclature. We noted the number of articles describing probands with that variant (excluding functional studies). Population frequency was defined from gnomAD (v.2.1.1) for worldwide frequency and RUSeq because it was not previously available for assessment (79, 80). We used these databases from July 2022 to November 2022. We did not use pathogenicity terms or copy the conclusions of the article. Pathogenicity status was reassessed using the Varsome database (81). In Varsome, pathogenicity status was determined according to the ACMG recommendations, taking into account the diagnosis, the presence of affected relatives, and the segregation of the variant in the family (75). Varsome also automatically takes into account ClinVar data, population frequency data, and predictive algorithms (82). If the segregation of the variant in a family was partial, we used the “Unknown” option in Varsome. The pathogenicity status and the date of accession to the Varsome and ClinVar databases were reported in the Gene_variants tables.

### 2.5. Quality control

Variants NC_000010.11:g.31319149_31319182delinsgggaggggtggaggcggaggggtGGGGGGGAAGG, NC_000010.11:g.31319183_31319189delinsGGGAGGG, NC_000010.11:g.31319190_31319193delinsAGGG from the article Tang H. et al. were not included in the review because checkup in Mutalyzer assigned them as reference sequences (83).

To control our variant classification methods, we performed the same process of search, data extraction, variant description, and synthesis of results for ZEB1 variants in PPCD, as ZEB1 null variants were confirmed to be pathogenic in PPCD subtype 3.

### 2.6. Synthesis of the results

If at least one article reported that the gene was expressed in the transcriptome of *ex vivo* corneal endothelial samples, the gene was considered to be expressed in the corneal endothelium.

For each variant reported in FECD in the SLC4A11, ZEB1, LOXHD1, and AGBL1 genes, we assessed the pathogenicity status according to the ACMG criteria implemented in the Varsome tool, taking into account diagnosis, segregation data, and presence of affected relatives. If there was information that there were familial cases where this variant was studied, this was indicated in the Varsome input window as the presence of affected relatives. Non-segregation was indicated in the Varsome input window only if another family member with FECD did not have the evaluated variant (phenotype +, genotype -). Incomplete penetrance and age-dependent non-penetrance in family members were not counted as non-segregation. If the variant was observed in a case with a clear alternative genetic cause of the disease, the BP5 criterion was added to the criteria defined by Varsome. If the population frequency from RUSeq was higher than >1%, the BA1 criterion was added to the criteria defined by Varsome. Varsome also takes into account published functional studies, so we checked whether the results of the functional studies were included in the evaluation. If not, criteria accounting for the results of the functional analysis (PS3 or BS3) were added to the criteria defined by Varsome. The absence of gene expression data in the corneal endothelium was considered as a result of functional analysis and added to other available functional analysis results as BS3 criterion. No criteria (PS3 or BS3) were added when there were conflicting functional analysis results. The summarized pathogenicity status of the variant was entered in the corresponding Gene_variants column (Supplementary Table S1).

We carried out a meta-analysis for selected variants. We calculated MAF for these variants based on information from the articles. If control groups were included in the article, we also calculated the MAF in them. For variants without the described genotype, two allele frequencies were calculated:

- the maximum frequency, based on the assumption that all carriers are homozygous.
- the minimum frequency, assuming that all carriers are heterozygous.

Meta-analysis was carried out with the R packages Hmisc (v4.7-0) (84) and forest plot (v2.0.1; Gordon and Lumley, 2022) (85). 95% confidence intervals for allele frequencies were calculated as binomial proportion confidence intervals (exact binomial test) for the allele frequency of each variant from the article and gnomAD (v2.1.1) data. Allele frequencies and meta-analysis results were visualized using forest plots.

The number and frequency of variants grouped by pathogenicity status in each gene were estimated using only case–control studies and consecutive case series. Studies that did not report the number of probands harboring a variant (i.e., only MAF was reported) were excluded (28). Studies investigating a mixed group of corneal dystrophies where the number of probands with each diagnosis was not reported were also excluded (86).

### 2.7. Outcomes

We concluded that the causal role of the gene in the pathogenesis of FECD was not confirmed if none of the variants in this gene were classified as pathogenic or significantly associated with the phenotype.

### 2.8. Sources of potential bias

Because of the manual search, there is a potential bias in the selected articles, although it was conducted by three reviewers, one of whom conducted the search independently. In addition, data extraction was done manually, although the risk of errors was minimized by double-checking all data included. The genomic, coding, and amino acid sequence coordinates of each variant were validated for each variant by cross-search in dbSNP and ClinVar databases, Mutalyzer, and Varsome tools. To minimize personal bias in pathogenicity classification according to ACMG criteria, we used the automated tool Varsome.

## 3. Results

### 3.1. Search results

**Figure 1:**
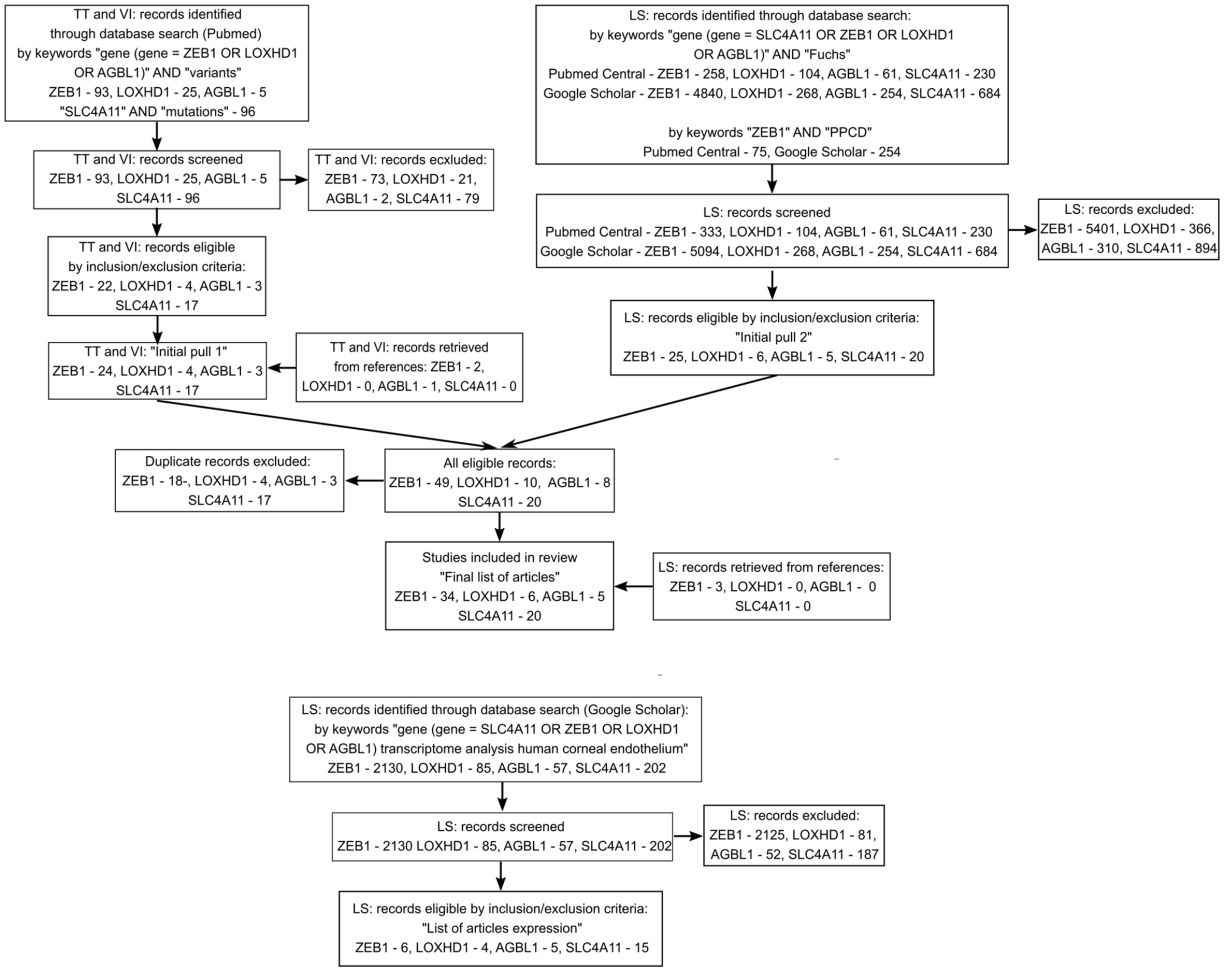
Flow diagram summarizing the screening method and study selection process.

A search in the PubMed, PubMed Central, and Google Scholar databases, as well as screening of reviews and references, resulted in the inclusion of 51 unique articles into the review of variants and 20 unique articles with data on transcriptome analysis of the corneal endothelium. The flow diagram of the search is presented in Figure 1. The full list of articles is shown in Supplementary Table S1. Twenty articles investigating SLC4A11 variants were included in the analysis (29, 43, 48, 53, 55, 56, 83, 87, 91–99, 119, 150, 151). ZEB1 variants in PPCD subtype 3 were extracted from 23 articles (40, 114–118, 120, 130, 132–136, 139–142, 152, 154, 156, 158–160). Fourteen articles provided information on ZEB1 variants in FECD patients (26, 40, 49, 53, 55, 56, 83, 116, 119, 120, 151, 153, 155, 157). LOXHD1 variants in FECD patients were analyzed in six articles (24, 28, 48, 55, 83, 157). AGBL1 variants in FECD were found in the five articles (25, 28, 48, 55, 86).

Reasons for exclusion of records on variants included: written not in English, conference paper, poster, review, not in Homo sapiens, and variants identified not in FECD or PPCD patients. Reasons for the exclusion of articles on transcription included: conference paper, review, not in Homo sapiens, gene expression evaluated in tissue other than corneal endothelium, gene expression was evaluated by PCR or Western blotting.

### 3.2. SLC4A11 variants in FECD

We extracted and entered data on 64 unique SLC4A11 variants in FECD cases. Table 1 summarizes the types and pathogenicity status of the reported variants. SLC4A11 variants were mostly investigated by sequencing all exons and splice sites of the SLC4A11 gene or by genotyping selected SNVs in case series or case–control studies (Supplementary Table S1). For the first time, four missense and one frameshift SLC4A11 variants in the heterozygous state were described by Vithana et al. in a cohort of Chinese and Indian FECD patients (87). In the studies by Gupta et al., Okumura et al., Igo et al., and Skorodumova et al., no missense variants were found in SLC4A11 in FECD patients (48, 53, 55, 56). In the study by Okumura et al., only synonymous or intronic variants were detected (55). All synonymous and intronic variants were classified as benign or probably benign (Supplementary Table S1).

**Table 1:** Summarized types and pathogenicity status of the reported variants in SLC4A11, ZEB1, LOXHD1, and AGBL1 variants in PPCD3 and FECD.

Gene		SLC4A11	ZEB1		LOXHD1	AGBL1
Disease		FECD	PPCD3	FECD	FECD	FECD
Classifier	Unique variants	64	63	42	26	4
By type	Gross deletions	0	5	0	0	0
	Upstream variants	0	2	0	0	0
	Frameshift variants	2	29	0	0	0
	Nonsense variants	3	14	1	0	1
	Splice-site variants	0	3	0	0	0
	Missense variants	23	1	15	19	2
	Start loss variants	0	2	0	0	0
	Synonymous variants	14	2	7	0	0
	Intronic variants	20	5	18	5	1
	3’ Untranslated region variants	2	0	0	1	0
	Intergenic variants	0	0	1	1	0
By inheritance	Familial cases	24	43	2	3	1
	Sporadic cases	40	20	40	23	3
By impact	Pathogenic	7	42	0	0	0
	Likely pathogenic	11	11	1	0	0
	Uncertain significance (VUS)	7	0	5	4	2
	Likely benign	13	2	9	14	1
	Benign	26	8	27	8	1

Table 2 shows the total number of probands with variants identified in the consecutive case series and case–control studies, grouped according to their pathogenicity status. VUS, likely pathogenic or pathogenic variants were detected in 2.5% (17/675) of all genotyped FECD probands.

**Table 2:** Number of FECD probands with variants identified in studies with consecutive series design and case–control studies grouped by the pathogenicity status.

Gene	SLC4A11	ZEB1		LOXHD1	AGBL1
Disease	FECD	PPCD3	FECD	FECD	FECD
Total genotyped probands	675	125	736	400	136
Pathogenic	1	27	0	0	0
Likely Pathogenic	10	3	1	0	0
VUS	6	0	4	4	1
Likely benign	70	0	10	56	0
Benign	50	23	100	18	0

Some carriers of the variants identified in the case–control studies had a family history (29, 87). A three-generation family was described by Riazuddin et al. (29). The NP_114423.1:p.Gly742Arg variant was detected in all three affected members. One member who was too young to be affected also carried this variant. All other unaffected members had reference alleles, so this variant segregated with the phenotype in all members old enough to have FECD symptoms. In the case report of a large multigenerational family described by Tang et al., no SLC4A11 variants were found to segregate with the phenotype (83). Of note, only synonymous and intronic variants were detected in this family.

SLC4A11 missense and loss-of-function variants in the homozygous state have been reported to cause congenital hereditary endothelial corneal dystrophy type 2 (CHED2) (88–90). In most cases, parents of CHED probands carry heterozygous pathogenic variants. Therefore, researchers investigated whether parents of CHED probands with defined SLC4A11 variants have FECD. Such an analysis was performed in two studies: Kim et al. described one family and Chaurasia et al. described eight families (91, 92). At least one parent in each family had cornea guttata, although they were clinically asymptomatic, including a 62-year-old mother in a family described by Kim et al. The mean age of the parents in the study by Chaurasia et al. was 32.5 years, so they were too young to have late-onset FECD manifestation. The Krachmer score or endothelial cell density for the 62-year-old mother in the study by Kim et al. was not available to assess the diagnostic criteria. The authors concluded that parents of children with CHED are at risk of developing FECD. We considered that it is impossible to estimate the segregation of variants with FECD phenotype in studies by Chaurasia et al. and Kim et al.

In summary, of 28 missense and loss-of-function SLC4A11 variants reported in FECD, 11 were found in families. Only in two familial cases, the family members have clinically manifested FECD, and only in one case, there was a segregation of the variant with the phenotype.

The effect of mutations associated with FECD and CHED2 on the SLC11A4 functionality has been extensively studied in cell culture models and *in silico*. Functional analyses were already performed in the first studies by Vithana et al. and Riazuddin et al. reporting SLC4A11 variants in FECD (29, 87). HEK293 cells were transiently transfected with the mutant and wild-type (WT) SLC4A11 cDNAs, respectively. Immunoblots showed that the immature form (monomer) was the predominant species of SLC4A11 mutants harboring p.Glu167Asp, p.Trp240Ser, p.Arg282Pro, p.Glu399Lys, p.Thr434Ile, p.Ser489Leu, p.Gly583Asp, p.Gly709Glu, and p.Thr754Met variants. Cell surface assays and immunolocalization results led to the conclusion that products of the SLC4A11 gene with the above-mentioned variants are predominantly accumulated inside the cell (retained in the endoplasmic reticulum) and are virtually absent on the cell surface (29, 87, 93). In a cell model that tested the correction of misfolding in the Gly709Glu-SLC4A11 mutant, glafenine was shown to restore trafficking and water flux activity at the cell surface (93).

Co-expression of the WT-SLC4A11 vector and vectors carrying SLC4A11 with FECD-associated variants (FECD-SLC4A11) did not lead to the restoration of dimer transport to the cell surface (94). Furthermore, the water flux function was significantly reduced. Cells co-expressed with Gly709Glu-SLC4A11 and WT-SLC4A11 had only 27 ± 2% of WT rate of cell swelling (95). The authors concluded that the absence of FECD-SLC4A11 mutants on the cell surface, even in the presence of WT-SLC4A11 expression, explains the nature of the autosomal-dominant inheritance type of FECD in patients harboring these variants (94). When the WT-SLC4A11 was co-expressed with SLC4A11 coding vectors carrying mutations associated with CHED (CHED-SLC4A11), partial recovery of SLC4A11 dimers transport to the cell surface was observed. The authors speculate that the partial recovery of dimers transport to the cell surface in the presence of WT-SLC4A11 expression may explain the cause of the autosomal recessive inheritance of CHED.

Li with co-authors studied the functional effects of some above-mentioned variants in the hamster fibroblast (PS120) cell line that lacks the Na + -H+ exchanger (NHE) (96). The results for the Trp240Ser-SLC4A11 mutant were in contrast to those obtained in the HEK293 cell model, as they indicated that it reaches the cell membrane. The results on the surface trafficking of the Val507Ile-SLC4A11 mutant were consistent with the results reported by Soumittra et al. Li with co-authors confirmed reduced NH3-sensitive electrogenic H+ transport activity in Trp240Ser-SLC4A11 and Val507Ile-SLC4A11 mutants.

Some variants (p.Val507Ile, p.Tyr526Cys, p.Val575Met, p.Ser565Leu, and p.Gly834Ser) did not cause the reduction of total SLC4A11 in a cell model, and the amount of mature form was indistinguishable from WT-SLC4A11 (29, 43, 97, 98). Confocal immunolocalization was consistent with Western blotting results: mutants carrying p.Val507Ile, p.Tyr526Cys, p.Val575Met, and p.Gly834Ser variants were mostly located at the cell membrane with some cytoplasmic fraction (29, 43). In the water flux assay, mutants caring p.Tyr526Cys, p.Ser565Leu, or p.Val575Met variants were shown indistinguishable from WT-SLC4A11 or slightly reduced functionality (43, 98). This led to questioning the pathogenic mechanism of these variants.

Analysis of these variants in a three-dimensional model of SLC4A11 protein revealed that 526, 565, and 575 residues were located in an extracellular loop 3 (EL3) (98, 99). In the SLC4A11 model, p.Val575Met and p.Val507Ile were predicted to result in a lack of symmetry at the close contact point between the subunits and alteration of the dimeric interface (99). No deleterious structural change induced by the p.Gly834Ser variant was found in a SLC4A11 model (99). The presence of four FECD-associated variants in EL3 suggested the involvement of EL3 in cell adhesion (98). HEK293 cells were transfected with vectors carrying SLC4A11 with p.Tyr526Cys, p.Ser565Leu, or p.Val575Met variants. A significant reduction was observed in a cell adhesion assay (98). Additional experiments with cultured CEnCs transfected with the SLC4A11-EL3 transmembrane-GPA integrated chimera confirmed the role of EL3 in CEnC adhesion. The authors concluded that the pathogenic effect of variants in EL3 could be explained by the defective adhesion of CEnCs to the Descemet membrane and their subsequent detachment.

The SLC4A11 expression in the transcriptomic data of corneal endothelial samples and cell cultures was reported in as many as 18 studies as SLC4A11 is specifically expressed in the corneal endothelium (Supplementary Table S1) (100–104). These included studies using *ex vivo* corneal endothelial samples (100–104, 106–109, 161). Expression of SLC4A11 has been reported in fetal and adult tissues (101). It was also expressed in H9 human embryonic stem cell-derived corneal endothelium, induced pluripotent stem cell-derived corneal endothelium, human corneal endothelial progenitor cells, differentiated human corneal endothelial progenitor cells, primary cultures of the corneal endothelium, and the corneal endothelial cell lines HCEnC-21 T, HCEC-12, and HCEC-B4G12 (100, 102, 105, 109–111). Frausto et al. (112) reported decreasing in SLC4A11 expression level with passages. SLC4A11 total expression was upregulated in samples of FECD patients (69, 113).

In summary, transcriptomic data from studies confirm SLC4A11 expression in the corneal endothelium. Functional analyses support the pathogenicity of several missense, nonsense, and frameshift variants. Two pathogenic mechanisms of missense variants were described: reduction of NH3-sensitive electrogenic H + -transport activity and impaired adhesion capacity. Segregation of SLC4A11 variants with the FECD phenotype has been reported in one family.

### 3.3. ZEB1 variants in PPCD3

We extracted and entered the data on 63 unique ZEB1 variants in PPCD3 cases. In one article among 14 tested probands with PPCD3, no ZEB1 variants were identified (40). Summarized types and pathogenicity statuses of reported variants are presented in Table 1. The most common variant reported in PPCD3 was NM_030751.6: c.1576dup. It was identified in five probands (Supplementary Table S1). Fifty of 62 reported PPCD3 probands with variants in exons, splice sites, and gross deletions had information on phenotype and/or genotype in the family. This resulted in 41 cases when segregation information was available. In most familial cases, variant had full segregation with the phenotype, in one case – partial (114). There was one reported missense variant NP_110378.3:p.His157Asp in a patient that also harbored a loss-of-function variant, so the pathogenicity status was rated as likely benign (115). Two synonymous variants were evaluated as benign. All other exonic variants were rated as pathogenic or likely pathogenic.

The frequency of pathogenic or likely pathogenic ZEB1 variants in the included consecutive case series and case-control studies was estimated to be 24% (30/125, Table 2).

Functional analyses of 16 variants were available from the included records (115–118). Chung et al. in an HCEnC-21 T cell model with transient transfection investigated functional consequences of 10 frameshifts and three nonsense mutations identified in PPCD3 patients (116). All mutations caused the truncation of the protein, and some mutations affected localization in the cell. Dudakova et al. assessed pre-mRNA splicing in transcript harboring NM_030751.6:c.482–2A > G splice-site variant using blood RNA (115). They showed that this variant causes exon 5 skipping and insertion of a premature termination codon. Chung et al. carried out transcriptional profiling of the cornea sample from the PPCD3 patient with NM_030751.6:c.1381delinsGACGAT variant in ZEB1 (117). Although differential gene expression analysis was limited by the small number of samples and their heterogeneity, authors observed a 6.7-fold decrease in the corneal endothelial ZEB1 in a PPCD3 patient with the frameshift variant. Immunohistochemical analysis of the cornea sample of the patient with NM_030751.6:c.1613del variant indicated aberrant activation of canonical Wnt signaling (118). Thus, functional analyses of 16 variants confirmed the pathogenic impact of ZEB1 loss-of-function variants in PPCD3 pathogenesis.

### 3.4. ZEB1 variants in FECD

A total of 14 studies were included in which ZEB1 variants were investigated in FECD cases. Forty-two unique variants have been identified in FECD cases. In one article, none of the five genotyped variants were found in 36 probands (55). The summarized types and pathogenicity status of the reported variants in FECD cases are shown in Table 1. Only one ZEB1 null variant was reported in FECD, and it was classified as likely pathogenic. Of the 15 missense variants, five were classified as VUS, five were classified as likely benign, and five were classified as benign. The frequency of ZEB1 VUS or likely pathogenic variants in the included consecutive case series and case–control studies was estimated to be 0.6% (5/736).

**Figure 2:**
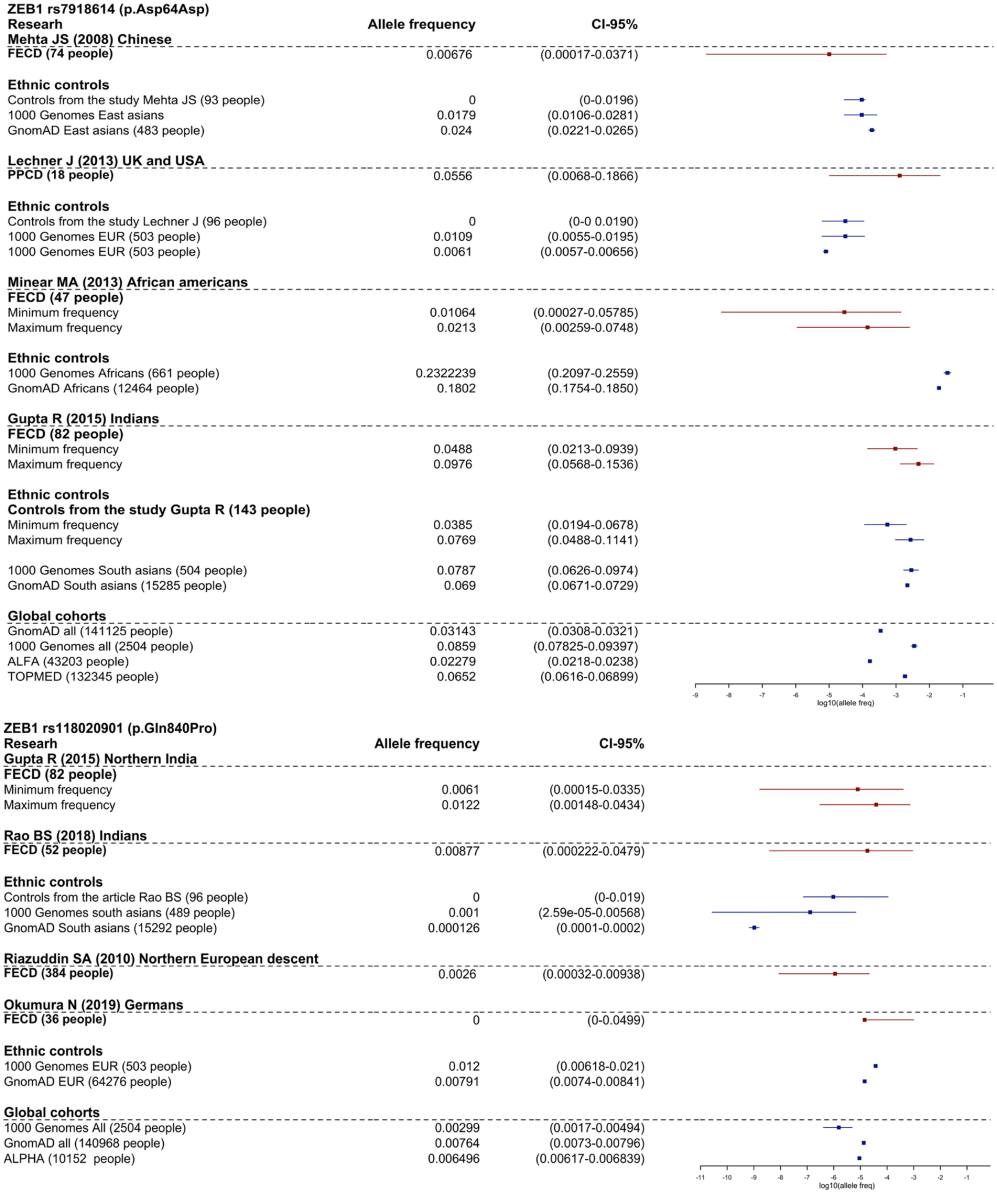
Frequency allele distribution of rs118020901 and rs7918614 minor alleles (95% confidence interval). The red box plots indicate the frequency in the affected groups, and blue boxplots show allele frequency in control groups from the studies and from population frequencies databases. The *X*-axis is log10 values of allele frequencies. For cases where the calculation of allele frequencies was impossible, we applied two assessments: up estimation point – assumption that all carriers are homozygotes for the variant, low estimation point - all carriers are heterozygotes for the variant.

Four studies investigated the association of ZEB1 variants in FECD and control groups, but none found a significant association (49, 56, 53, 119). Variants NP_110378.3:p.Asp64Asp (rs7918614) and NP_110378.3:p.Gln840Pro (rs118020901) were investigated in more than two cohort studies included in the review. We presented meta-analyses of the minor allele frequencies reported in the articles and gnomAD (v.2.1.1) frequencies in Figure 2.

As shown in Figure 2, the frequency of rs7918614 minor allele is higher in African population controls (gnomAD (v.2.1.1) and 1,000 Genomes) than in FECD patients. The risk allele rs118020901 does not reach significance compared to controls in any group.

It is interesting to note that NP_110378.3:p.Gln840Pro has an allele frequency of 1.11% in RUSeq cluster 1 (European part of Russia) and 1.33% in RUSeq cluster 3 (Siberian and Far Eastern parts of Russia), which is almost two times higher than the total gnomAD (v2.1.1) allele frequency of 0.76%. This did not support the pathogenicity of this variant.

Among the reported cases with ZEB1 variants, only two had familial phenotype and/or genotype information. No non-reference ZEB1 variants were found in probands in the multigenerational family described by Tang et al. (83). Therefore, this study was not included in the ZEB1_familial_cases table. One variant was identified in a familial case of keratoconus (120). FECD was diagnosed only in the mother of the proband. The proband did not have FECD at the time of molecular analysis. As the proband could not be excluded to have FECD in later years, we could not conclude the segregation of the variant with the phenotype. Thus, segregation information for the ZEB1 variants was only available in one FECD family (26). The segregation of the ZEB1 variant with the phenotype was partial. The NP_110378.3:p.Gln840Pro variant was absent in two family members diagnosed with FECD who were over 50 years of age. No FECD families with full segregation of ZEB1 variants were found.

Functional analysis of ZEB1 missense variants discovered in FECD patients has been reported in three articles (26, 116, 120). While Chung et al. did not find any effect of six missense variants on ZEB1 protein abundance, molecular size, or intracellular localization in a HCEnC-21 T cell model, Riazuddin et al. found an effect of two variants in an *in vivo* zebrafish embryo model (26). An antisense translation blocking morpholino suppressed the translation of zebrafish tcf8. Injection of RNA encoding WT ZEB1 rescued the phenotype of the embryos. RNA encoding mutant ZEB1 variants p.Asn696Ser, p.Pro649Ala, p.Ala905Gly, and p.Gln840Pro rescued the phenotype of the embryos, so that they were almost indistinguishable from embryos injected with WT ZEB1 RNA. Injection of RNA harboring variants p.Asn78Thr and p.Gln810Pro only partially rescued the phenotype of the embryos. Thus, they may have some effect on ZEB1 functionality. However, p.Asn78Thr was classified as benign based on BA1 criterion: allelic frequency higher than 5% in the African gnomAD (v.2.1.1) population and homozygosity in 30 exomes and genomes.

Lechner et al. investigated the effect of the His640Pro variant found in patients with FECD and keratoconus on cultured keratocytes (120). Dysregulation of COL4A1, COL4A2, COL4A3, COL4A4, and COL8A2 gene expression was demonstrated.

The ZEB1 expression in the transcriptomic data of corneal endothelial samples and cell cultures was reported in 10 studies (Supplementary Table S1). Its expression was detected in pediatric, young, and adult *ex vivo* corneal endothelial samples (100, 103, 108, 109). In one study, no ZEB1 expression was detected in *ex vivo* corneal endothelium from old donors (100). ZEB1 was expressed in H9 human embryonic stem cell-derived corneal endothelium, induced pluripotent stem cell-derived corneal endothelium, primary cultures of the corneal endothelium, and the corneal endothelial cell lines HCEnC-21 T, HCEC-12, and HCEC-B4G12 (100, 105, 109, 112). ZEB1 was reported to have a low but confident expression (112). It was not differentially expressed in corneal endothelial samples from FECD patients (113). However, its expression was decreased in corneal endothelial samples from PPCD patients (117, 118). ZEB1 was also a differentially expressed gene in *ex vivo* bullous keratopathy samples compared to control corneal endothelial samples (121).

To summarize, analysis of studies with the transcriptomic data confirmed ZEB1 expression in the corneal endothelium. The functional analysis did not support the pathogenicity of p.Asn696Ser, p.Pro649Ala, p.Ala905Gly, and p.Gln840Pro variants, whereas p.Gln810Pro and p.His640Pro may have some effect on ZEB1 functionality. ZEB1 variant segregation with the FECD phenotype was reported only in one family and was partial.

### 3.5. LOXHD1 variants in FECD

Twenty-six unique LOXHD1 variants have been identified in FECD cases. In the study by Okumura et al. in a cohort of 36 probands, no variants were detected among three genotyped variants (55). Furthermore, Skorodumova et al. did not find any carriers of rs113444922 minor alleles in a cohort of 100 FECD patients (48). The summarized types and pathogenicity status of the reported variants in FECD cases are shown in Table 1. No variants were classified as pathogenic or likely pathogenic. Of 19 missense variants, four were classified as VUS, 12 variants were classified as likely benign, and three variants were classified as benign. Although three variants were detected in familial cases, only p.Arg547Cys segregated at least partially with the phenotype. Linkage analysis using STR markers in the study by Riazuddin et al. showed that one family member was diagnosed with FECD but did not have the p.Arg547Cys variant (24). However, there was a locus that all affected members had and all unaffected members did not. This locus is between probes D18S484 and D18S1152, which define a region NC_000018.10:g.54211458–57049354 of the 18th chromosome. This region does not contain the LOXHD1 gene, but it does contain the known FECD-associated gene – TCF4.

Functional analysis was conducted only for the p.Arg547Cys variant (24). Immunofluorescence (IF) staining of LOXHD1 protein in corneal samples of FECD patients with variant, FECD patients without variants, and control corneal samples showed the effect of the variant on protein localization. In addition, cells transfected with the plasmid encoding GFP-tagged mutant LOXHD1 showed distinct cytoplasmic puncta compared with cells transfected with the plasmid encoding GFP-tagged WT LOXHD1 (24).

The results of the transcriptomic analysis in four articles showed the absence of LOXHD1 expression in *ex vivo* corneal endothelial samples, contradicting the findings of Riazuddin et al. results (31, 100, 108, 109). No LOXHD1 expression was detected in H9 human embryonic stem cell-derived corneal endothelium, induced pluripotent stem cell-derived corneal endothelium, primary cultures of the corneal endothelium, and the corneal endothelial cell lines HCEnC-21 T, HCEC-12, and HCEC-B4G12 (100, 105, 109). In corneal endothelial samples from FECD patients, no LOXHD1 expression was observed (31).

In conclusion, the results of IF staining are inconsistent with the absence of the LOXHD1 gene expression in the transcriptomic data of corneal endothelial samples. The absence of LOXHD1 expression in corneal endothelium makes it impossible to synthesize protein and detect the effect of the variant.

### 3.6. AGBL1 variants in FECD

Four unique AGBL1 variants have been reported in FECD patients (Table 1). For AGBL1 variants reported in FECD, VUS was the highest pathogenicity score. There were two such variants. The study by Riazuddin et al. was the first to report an association of AGBL1 with FECD (25). A combination of linkage analysis and target sequencing was used to search for a causal mutation at a locus on chromosome 15 in a family of 12 individuals with FECD and four healthy family members. A nonsense variant NP_689549.3:p.Arg1074* in the AGBL1 gene was identified as a candidate variant. The authors stated that the mutation, which was present in eight of the 12 affected family members, segregated with disease in the family under a multilocus model. A thorough analysis of the metadata and pedigree chart from the article resulted in two of the affected family members having trace signs of FECD and another five having <=2 points on the Krachmer scale. Three affected members with FECD (>=1 Krachmer score) had reference genotypes (II-1, II-2, and III-4). One unaffected member carried NP_689549.3:p.Arg1074* variant (III-3). Segregation of the FECD phenotype with the nonsense variant genotype in family members was only partial. To date, this is the only familial FECD case in which the AGBL1 variant has been reported.

The NP_689549.3:p.Cys1036Ser variant was detected in sporadic FECD cases (25, 48). The rs118086539 variant was reported to have a modest association in GWAS, but did not reach the genome-wide significance threshold (28). NP_689549.3:p.Arg794His and NP_689549.3:p.Arg1074* variants were identified in patients with atypical corneal dystrophy, which were defined as FECD based on the detection of this variant (86).

Functional analysis was carried out for the NP_689549.3:p.Arg1074* and NP_689549.3:p.Cys1036Ser variants (25). In an NIH 3 T3 cell model, transient transfection of the vector encoding the mutant protein resulted in decreased protein abundance, while localization did not change. The presence of AGBL1 protein was detected by IF staining of the patient’s cornea (25). Serial analysis of gene expression (SAGE) also detected AGBL1 expression (122). However, transcriptomic analysis of donor and FECD corneal endothelium samples in four studies showed no AGBL1 expression (31, 100, 108, 109).

Overall, the results of IF staining of the cornea and SAGE conflict with the absence of AGBL1 expression in corneal endothelium according to RNA-seq results. The absence of AGBL1 expression in corneal endothelial cells makes it impossible to synthesize protein and detect the impact of the variant.

## 4. Discussion

The SLC4A11 gene encodes a protein that is a member of the Solute Carrier 4 (SLC4) family of bicarbonate transporters (previously known as BTR1, NaBC1). However, it has been shown to be a Na+-dependent OH-(H+) and NH3+-dependent H+ transporter (123, 124). Ion transporters allow the endothelial cells to function as a barrier between the aqueous humor of the anterior chamber and the dehydrated corneal stroma, so the causal role of pathogenic variants in SLC4A11 in corneal dystrophies is not surprising. Vithana et al. first reported that pathogenic variants in SLC4A1 cause congenital hereditary corneal dystrophy 2 (CHED2). CHED2 is an autosomal recessive disease caused by homozygous variants in SLC4A11 (88). Clinically, it is characterized by bilateral diffuse corneal opacities (typically “ground glass” appearance) with corneal endothelium bedewing without associated corneal vascularization. In most cases, symptoms appear in infancy. However, delayed onset CHED is also possible (125). The disease is prevalent in populations where consanguineous marriages are common (89, 126, 127). Homozygous variants in SLC4A11 also cause Harboyan syndrome (128). This rare syndrome is characterized by corneal dystrophy and perceptive deafness. Because CHED2 and FECD are both endothelial dystrophies and share some common features, such as Descemet membrane thickening, it has been suggested that they may be caused by different variants in the same genes. Indeed, mutation screening in a cohort of Indian and Chinese patients with FECD revealed heterozygous variants in 4.5% of cases (87). Later, nine more case–control and case series studies of variants in SLC4A11 were investigated. Some studies have identified only synonymous or intronic variants that have been classified as benign or likely benign. Thus, the prevalence of VUS, likely pathogenic or pathogenic SLC4A11 variants among FECD patients is low (2.5% in case–control studies and consecutive case series) according to our analysis. Nevertheless, evidence for the pathogenic effects of missense SLC4A11 variants has been obtained from family cases and functional studies.

Many functional studies of variants have been performed to provide evidence for the pathogenic effect of variants in SLC4A11. Two main mechanisms have been discovered: by affecting the SCL4A11 transporter activity and its adhesion function (96, 98). They provided an interesting theory to explain the difference between variants causing CHED2 and FECD. FECD missense variants affect protein function so that the mutant protein has only 6–36% of the functional activity of the WT protein, and the presence of the WT protein does not improve its activity. Missense variants found in CHED2 affect the protein, but the mutant protein can perform its function at a level of 33–41% of the WT when it forms dimers with the WT protein (94, 95). In other words, individuals carrying these variants in the heterozygous state may not manifest the disease. This is consistent with the results of Kim et al. who found guttae in the mother of the proband with CHED2, but no subjective symptoms of FECD (91). In addition, other studies describing SLC4A11 variants in CHED2 probands did not report FECD in parents or grandparents. The presence of FECD in heterozygous carriers of CHED2 variants in SLC4A11 should be further investigated in additional multigenerational families with members older than 50 years and careful grading, pachymetry, and specular microscopy data.

To conclude, although the prevalence of missense SLC4A11 variants in FECD patients is low, there is sufficient information on segregation in families and functional results to classify some of them as pathogenic, thus confirming their role in the pathogenesis of FECD.

Posterior polymorphous corneal dystrophy is a rare autosomal-dominant endothelial dystrophy characterized histologically by the transformation of endothelial cells into epithelial-like cells. PPCD clinical signs include vesicles, bands, and geographic opacity of the posterior corneal layers, as well as iridocorneal adhesions, iris atrophy, pupil ectropion, and retrokeratic membranes (129). Genetic heterogeneity has been demonstrated in PPCD.

Previous studies have shown that loss-of-function variants in the ZEB1 gene are involved in the development of PPCD type 3 (115). Our analysis of consecutive case series and case–control studies showed that 24% of patients with PPCD harbor pathogenic or likely pathogenic variants in ZEB1. Pathogenic variants in two other epithelial-associated transcription factors that repress ZEB1 transcription, ovo-like 2 (OVOL2) and grainy head-like transcription factor 2 (GRHL2), are known to cause PPCD types 1 and 4, respectively (118, 130, 131).

Patients with PPCD3 have been reported to have non-ocular phenotypes. These include inguinal hernias and corpus callosum (114, 132–135). The age of PPCD3 manifestation varies from childhood to the third decade of life (114, 129). PPCD3 shows significant phenotypic variability, including intrafamilial, with incomplete penetrance (114, 136). Some carriers of pathogenic variants can be asymptomatic (137).

ZEB1 gene encodes zinc finger E-box binding homeobox 1 transcription factor, also known as TCF8 (transcription factor 8). This transcription factor plays a role in epithelial–mesenchymal transition (EMT) by inhibiting the expression of E-cadherin 1 (encoded by CDH1). ZEB1 is expressed in a variety of cells, including neural cells, immune cells, mesenchymal cells, and corneal endothelial cells. ZEB1 has been shown to play an important role in the cornea, regulating differentiation, wound healing, neovascularization, and production of extracellular matrix (138).

At first, the association between ZEB1 and PPCD3 was shown by Krafchak and colleagues (114). They revealed a frameshift variant (NM_030751.6:p.Gly973ValfsTer14) in a family with the PPCD3 history. This mutation showed full segregation with pathogenic phenotype and caused changes in the ZEB1 protein structure. In recent years also gross deletions were detected in PPCD3 patients (115, 139, 140). Almost two-thirds of the variants have been described in family cases with information on segregation (41/63). Some of them were confirmed *de novo* loss-of-function variants (114, 115, 135, 141, 142). The functional analysis confirmed the pathogenic effect of 16 loss-of-function variants. All of these findings supported the causal role of loss-of-function variants in ZEB1 in the development of PPCD3. A systematic review of ZEB1 variants in PPCD3 development was performed to control the quality of our methods. As the known role of ZEB1 variants in PPCD3 development was confirmed, this provides evidence for the adequacy of our methods.

The presence of candidate variants in the ZEB1 gene was investigated in FECD cases. Although studies of variants in the ZEB1 gene have genotyped more patients with FECD than studies of SLC4A11, fewer VUS or potentially pathogenic variants, and no pathogenic variants have been identified (0.6 and 2.5%, respectively). Likely pathogenic variant status was assigned to the single nonsense mutation found in a FECD patient. All other exonic variants were missense or synonymous. The detection of the ZEB1 nonsense variant in a patient with FECD is unusual. It would be desirable to investigate this case in more detail. Less likely, it is related to misdiagnosis (the patient has PPCD3). Another possible explanation could be the asymptomatic carriage of the loss-of-function variant, as was shown in the study by Dudakova et al. (137). In this case, the exclusion of the presence of the CTG18.1 expansion would be very helpful.

No FECD families with the full segregation of ZEB1 were found (26, 120). Therefore, in further analysis of variants in ZEB1 in patients with FECD, it would be highly valuable to include first-degree relatives in the study and to perform a comprehensive ophthalmic examination and genotyping.

Functional analysis of missense variants in cell line models did not support the pathogenicity of these variants (116). Analysis using an *in vivo* zebrafish embryo model detected the pathogenic effect of two missense variants (26). This model reflects the effect of the variant in the homozygous state, which cannot quite be transferred to its effect in the heterozygous state. Thus, these results should be treated with caution. To further investigate the action of missense variants in ZEB1, it would be desirable to use other functional methods, such as the creation of cell models; transcriptome analysis, chromatin immunoprecipitation coupled with high-throughput sequencing (ChIP-seq) or electromobility shift assay in cultured patient CEnCs or cell models (131, 143).

In summary, there was insufficient information on the segregation of variants in familial cases or functional analysis results to classify at least one variant as pathogenic. Thus, the causal role of the ZEB1 gene in the pathogenesis of FECD could not be confirmed. Aldave with coauthors has already questioned the role of ZEB1 in FECD (144).

Lipoxygenase homology domain 1 is a protein encoded by the LOXHD1 gene. It is conservative among vertebrates and consists of PLAT domains. LOXHD1 probably is involved in targeting proteins in the plasma membrane (145).

An analysis of the available literature on the LOXHD1 gene showed that the association between LOXHD1 and FECD was first reported in 2012 by Riazuddin et al. (24). Linkage analysis using STR markers in that study showed partial segregation of the p.Arg547Cys variant: one family member was diagnosed with FECD but did not have this variant.

The p.Arg547Cys variant is localized in the exon of the longest LOXHD1 isoforms, isoforms 1 and 6; in other isoforms, it is located in the 5′ upstream region. This means that the expression of isoforms 1 or 6 is necessary for the manifestation of the missense variant p.Arg547Cys. Several studies reported the absence of LOXHD1 expression in corneal endothelial transcriptomes (31, 100, 105, 108, 109). According to the expression database GTex,^1^ the long isoforms are not expressed in any tissue.

Transcriptomic data contradict the results of LOXHD1 protein staining in cornea samples by Riazuddin et al. LOXHD1 aggregates were found in the corneal endothelium and in the Descemet membrane of the FECD patient with the p.Arg547Cys variant. Specific protein detection without RNA expression is unlikely. Therefore, we checked the antibody which was used for IF staining: sc-85038 (Santa Cruz, USA). The description of this antibody states that the observed molecular weight of LOXHD1 protein in positive controls, IMR-32, Jurkat, and K-562 cells is 150 kDa. However, a Western blotting image shows a band between 90 and 132 kDa. This band most likely corresponds to LOXHD1 isoform 3, not the long ones. There were no positive controls for the long isoforms. The detection of long isoforms with this antibody is not confirmed. This also does not support the interpretation of staining with this antibody as the detection of LOXHD1 long isoforms in the study by Riazuddin et al.

To summarize, there is currently no evidence for the expression of LOXHD1 in general, nor for the expression of its long isoforms in sufficient amounts in the corneal endothelium.

Expression of the LOXHD1 gene in humans may be restricted to a certain stage of development. Theoretically, this could explain the presence of at least some isoforms in the Descemet membrane (as was identified by IF in the Riazuddin et al. study) and its absence in the endothelium.

Going back to the family from the Riazuddin et al. study, there was one locus that all affected members had and all unaffected members did not. This locus contained the TCF4 gene, but it did not contain the LOXHD1 gene. At the time of the article submission, the association of the TCF4 gene with FECD was already known according to the GWAS results from the Baratz et al. study in 2010 (27). Riazuddin et al. investigated the allele segregation of the rs613872 variant in the TCF4 gene in the family, but it was not confirmed. However, at the time the article was submitted, two facts were not known:

- the trinucleotide expansion repeats are associated with FECD – Wieben’s et al.’s study was published in 2012 (57).
- individual may harbor expansion without rs613872 minor allele – this was first mentioned in 2019 by Okumura et al. for a German cohort (55).

Riazuddin et al. did not have the opportunity to doubt the possible irrelevance of the rs613872 variant, or to examine the repeat expansion among family members, at the time of article submission.

Our review of reported variants in LOXHD1 with respect to segregation in FECD families and data on LOXHD1 expression in corneal endothelium did not reveal any pathogenic variants. Therefore, we can conclude that the causality of LOXHD1 gene variants for FECD was probably initially incorrectly identified and no substantial arguments have been found to date.

ATP/GTP-binding protein-like 1 is a protein encoded by the AGBL1 gene. It is believed that this gene catalyzes the deglutamylation of polyglutamylated proteins. An analysis of the available literature on the AGBL1 gene showed that the association between AGBL1 and some phenotypes was reported for the risk of coronary artery disease, carotid plaque, specific learning disorders, and cognitive endophenotypes of schizophrenia (146–149). No association had been reported between these phenotypes and FECD.

An association between AGBL1 and FECD was first reported in 2013 by Riazuddin et al. in a multigenerational family (25). NP_689549.3:p.Arg1074* variant was also detected in the unaffected member and not in all affected members. Thus, the segregation of this variant with phenotype was partial. It is worth noting that the authors did not exclude the presence of a CTG18.1 expansion in affected members as a specific FECD-associated variant, which had already been identified in about two-third of FECD patients at the time of article submission (57).

Transcriptomic data from five studies showed no AGBL1 expression in CEnCs (31, 100, 105, 108, 109). This contradicts SAGE results. SAGE with positive AGBL1 expression generated by Gottsch et al. in 2003 contains 10-bp fragments (tags) (122). These tags are very short and very ambiguous for conclusive results.

The most harmful variant was classified as VUS, and characterized by at least incomplete penetrance, and at most by the absence of expression and conflicts with protein detection in the endothelium. According to our analysis, the contribution of the AGBL1 gene to FECD pathogenesis was not confirmed.

## 5. Conclusion

Our analysis confirmed the causal role of SLC4A11 variants in the development of FECD. The causal role of ZEB1, LOXHD1, and AGBL1 variants in FECD has not been confirmed. Further evidence from familial cases and functional analysis is needed to confirm their causal roles in FECD. Since approximately two-third of late-onset FECD cases are associated with CTG18.1 expansion, this cause should be excluded before investigating other pathogenic variants.

## Data availability statement

The original contributions presented in the study are included in the article/Supplementary material, further inquiries can be directed to the corresponding author.

## Author contributions

LS and ES contributed to the conceptualization, design, and methodology of the study. VI and TT performed the initial search of articles, organized tables of variants, and entered the extracted data. LS performed the independent article search. LS checked the entered data. TT and ES performed the RNA-seq analysis. TT performed visualization. TT and LS wrote the first draft of the manuscript. ES wrote sections of the manuscript. VI and ES edited the draft. LS acquired funding. All authors contributed to the manuscript revision, read, and approved the submitted version.

## Funding

This study was supported by Russian Science Foundation, grant No 22-75-10157, https://rscf.ru/en/project/22-75-10157/.

## Conflict of interest

The authors declare that the research was conducted in the absence of any commercial or financial relationships that could be construed as a potential conflict of interest.

## Publisher’s note

All claims expressed in this article are solely those of the authors and do not necessarily represent those of their affiliated organizations, or those of the publisher, the editors and the reviewers. Any product that may be evaluated in this article, or claim that may be made by its manufacturer, is not guaranteed or endorsed by the publisher.

## Footnotes

^1^https://gtexportal.org/home/gene/LOXHD1
